# Skin Barrier and Autoimmunity—Mechanisms and Novel Therapeutic Approaches for Autoimmune Blistering Diseases of the Skin

**DOI:** 10.3389/fimmu.2019.01089

**Published:** 2019-05-14

**Authors:** Natalie E. Stevens, Allison J. Cowin, Zlatko Kopecki

**Affiliations:** Regenerative Medicine Laboratory, Future Industries Institute, University of South Australia, Adelaide, SA, Australia

**Keywords:** skin barrier, autoimmunity, autoantibody, skin blistering diseases, therapy, pemphigus, pemphigoid, epidermolysis bullosa acquisita

## Abstract

One of the most important functions of the skin besides regulating internal body temperature includes formation of the barrier between the organism and the external environment, hence protecting against pathogen invasion, chemical and physical assaults and unregulated loss of water and solutes. Disruption of the protective barrier is observed clinically in blisters and erosions of the skin that form in autoimmune blistering diseases where the body produces autoantibodies against structural proteins of the epidermis or the epidermal-dermal junction. Although there is no cure for autoimmune skin blistering diseases, immune suppressive therapies currently available offer opportunities for disease management. In cases where no treatment is sought, these disorders can lead to life threatening complications and current research efforts have focused on developing therapies that target autoantibodies which contribute to disease symptoms. This review will outline the involvement of the skin barrier in main skin-specific autoimmune blistering diseases by describing the mechanisms underpinning skin autoimmunity and review current progress in development of novel therapeutic approaches targeting the underlying causes of autoimmune skin blistering diseases.

## Introduction

The stratified squamous epithelium of the human epidermis forms a continuous barrier against the external environment and impairments in epithelial adhesions lead to disorders characterized by significant morbidity and/or mortality (1). The hallmark feature of autoimmune blistering diseases (AIBDs) is the disruption of the intact skin barrier as a consequence of blistering and erosions caused by production of autoantibodies against structural proteins in the epidermis or at the epidermal-dermal junction. AIBDs generally occur in the elderly, and often have substantial clinical and immunopathological overlap and polymorphic clinical presentation which can make diagnosis challenging (2). Immunologically, these conditions are driven by humoral and cellular autoimmune responses directed against distinct target antigens and can be classed in three main groups including pemphigoid and pemphigus diseases as well as dermatitis herpetiformis (DH) (3).

Over the past four decades, our knowledge of the pathophysiology of AIBDs has been greatly advanced by demonstrating that passive transfer of antibodies against skin antigens can disrupt the skin barrier and induce blisters in experimental animals models with clinical, histologic, and immunopathogenic responses similar to those observed in human disease (1). Each AIBD is characterized by the presence of specific autoantibodies targeting distinct antigens in the epidermis or at the dermal-epidermal junction. Intraepidermal blistering found in pemphigus disorders are caused by autoantibodies targeting cadherin proteins in desmosomes; subtypes pemphigus vulgaris and pemphigus foliaceus are associated with antibodies against desmoglein (dsg)-3 and−1, respectively. In bullous pemphigoid (BP), autoantibodies target two hemidesmosome components BP180 and BP230; and in epidermolysis bullosa acquisita (EBA) patients have autoantibodies target type VII collagen anchoring fibrils. In DH patients, autoantibodies target tissue and epidermal transglutaminase (eTG) proteins (3) however recently a case was reported where autoimmune intraepidermal and subepidermal blistering disease coexisted with a patient who was reported to have autoantibodies to both desmoglein (Dsg) 1 and BP230 (4).

AIBDs typically present with generalized blister eruption associated with itch however atypical presentations are often encountered. For example, 20% of BP patients present with “non-bullous” presentations, while anti-p200 pemphigoid patients that normally present with tense blisters with erythematosus often show normal skin resembling BP. Additionally, epidermolysis bullosa acquisita, an autoimmune disease associated with autoantibodies against type VII collagen, has several phenotypes including a classical form that mimics dystrophic epidermolysis bullosa, an inflammatory form that mimics BP, or a form more similar to mucous membrane pemphigoid-like lesions (2).

Diagnosis of AIBDs relies on direct immunofluorescence microscopy studies and immunoserological assays (5, 6). Multiple mechanisms of skin barrier disruption and blister formation in AIBDs have been described: in pemphigus disorders steric hindrance (the direct inhibition of protein-protein binding by autoantibodies) and cell signaling events cause desmosomal instability, while complement and inflammatory cell activation mediated through Fc-signaling cause keratinocyte death and blister formation in pemphigoid and epidermolysis bullosa acquisita (7–9). Development of targeted therapies and management of affected patients is often challenging due to frequent relapses, lack of efficacy and number of adverse events (10, 11). Current standard treatment options rely on non-specific immunosuppression, highlighting the need for development of targeted therapeutic approaches (12, 13). In this review we will focus on skin barrier involvement in mechanisms underpinning autoimmunity and describe the latest approaches for development of targeted therapeutics for the treatment of AIBDs.

## Skin Barrier and Mechanisms Underpinning Autoimmune Skin Blistering

More than 2.5% of the world's population is affected by autoantibody driven autoimmune disease, including AIBDs (7). The principles of autoantibody generation and detection in AIBDs have been reviewed extensively (7). Technological advancement in the last two decades have allowed us to identify the sequence of specific nanostructural and functional changes in the skin barrier following the binding of autoantibodies and define critical pathways and processes responsible for autoimmune pathology (14). The pathogenesis of AIBDs can be divided into three phases: (i) the *induction* phase (loss of self-tolerance or the initiation of autoimmunity to the target antigen), (ii) the *maintenance* phase (maintained production of autoantibodies) and (iii) the *effector* phase (autoantibody-mediated tissue damage). Specific mechanisms relating to these phases have been described for AIBDs, including pemphigus disorders, BP, EBA, and DH.

## Induction of Autoimmunity Against Skin Antigens

There are multiple theories that explain how the loss of tolerance to self-antigens initially occurs and it is understood that the majority of AIBDs are a product of several aberrant processes which disrupt skin barrier homeostasis. Genetic factors play an important role, as specific skin blistering diseases have varying prevalence in different populations and inherited human leukocyte antigen (HLA) types are associated with autoreactivity to specific autoantigens (15). Multiple HLA alleles have been identified which are associated with pemphigus vulgaris (15, 16), BP (17, 18), and EBA (19, 20). Genetic susceptibility is not limited to HLA types, as pemphigus vulgaris has been associated with mutations in *ST18* (a gene encoding a pro-apoptotic transcription factor) in certain populations (21) and experimental models of EBA have identified non-HLA murine gene loci that confer susceptibility to disease development (22), however further studies are required to extrapolate these findings to clinical populations.

Cell damage has been proposed as a common “triggering factor” which causes development of pathogenic adaptive autoimmune reactions—cell damage due to surgical trauma (23), UV radiation (24), neurological disorders and other pre-existing conditions (25–29), viral infection (30–33), and radiotherapy (34–36) have all been associated with disrupted skin barrier function and development of AIBDs (37). Cell damage via necrosis or necroptosis releases a complex intracellular milieu into the extracellular space which serves as a source of sensitizing autoantigens (38); additionally cell death results in the release of damage associated molecular patterns which stimulate localized inflammation and wound healing processes (39, 40). Normal healing responses following trauma aiming to re-establish the skin barrier cause infiltration of dendritic cells and other antigen presenting cells which may also participate in autoimmune sensitization (41, 42) of AIBDs.

Epitope spreading is an inbuilt mechanism of the adaptive immune system that aids in protecting against changing pathogens (43), however spreading from pathogenic to autologous epitopes and molecular mimicry of similar epitopes may also contribute to the formation of AIBDs (44). Fogo selvage, an endemic form of pemphigus foliaceus found in Brazilian populations, is associated with a history of sand fly bites and characterized by autoantibodies against Dsg1. These autoantibodies have shown cross reactivity to proteins present in sand fly saliva (45), which may represent epitope spreading from foreign proteins to similar autoantigens. Epitope spreading is also thought to be involved in paraneoplastic (PNP) pemphigus (46) where tumor-associated antigens may become targeted in an effort to destroy the tumor, however similar antigens may also be shared by keratinocytes (47). PNP pemphigus is most commonly associated with lymphatic malignancies, including non-Hodgkin's lymphoma and chronic lymphocytic leukemia. These malignancies are associated with the production and release of cytokines which can lead to over-stimulation of humoral immunity and autoimmune reactions, including disruption of skin barrier and development of AIBDs. Findings of autoimmune skin blistering in carcinoma patients has fuelled speculation that these diseases may be triggered by an anti-tumor immune response (48–50), however further studies are required to determine the relationship between these findings (51, 52). Coeliac-disease associated skin blistering, known as DH, is caused by antibodies against gluten-induced digestive enzyme tissue transglutaminase which undergo epitope spreading to cross-react with epidermal transglutaminase (eTG) leading to the disruption of the skin barrier and subsequent skin blistering (53, 54). Epitope spreading may also contribute to the diversity of and disease progression of AIBDs, as epitope spreading to related autoantigens has been associated with atypical or altered disease presentations (53, 55, 56).

AIBDs have been associated with the use of certain drugs which trigger pathogenesis through a variety of mechanisms. One of the most well-described etiologies is BP in diabetic patients taking dipeptidyl-peptidase 4 (DPP-4) inhibitors (57–59) which present with antibodies against the mid-portion of BP180. It has been suggested that DPP-4 inhibition reduces plasmin production and alters BP180 cleavage, resulting in altered antigenicity of BP180 (60) which is supported by the finding that symptoms generally subside after drug discontinuation. The use of immune checkpoint inhibitors for cancer therapy has been linked to secondary development of AIBD including mucous membrane pemphigoid and BP (61, 62), likely a consequence of nonspecific immune activation.

## Maintained Autoantibody Production

Autoantibodies are often present in healthy individuals but are generally non-pathogenic IgM antibodies with low affinity present in low levels and do not alter skin barrier homeostasis. For autoantibodies to gain pathogenicity, class-switching to IgG or IgA subtypes, somatic mutation and increased production occur after exposure to self-antigen. Glycosylation and sialylation patterns on autoantibodies also contribute to pathogenicity and antibodies stimulated via T-cell interaction within germinal centers exhibit reduced sialyation patterns and are pro-inflammatory (63). In the context of AIBDs, the presence of self-antigen released by damaged keratinocytes may stimulate production of autoantibodies, which in turn bind to healthy tissue to stimulate skin barrier disruption and further tissue damage and promote more autoantibody generation in a self-perpetuating cycle. Continued production of pathogenic autoantibodies may be achieved by ongoing B cell activation and production of short-lived plasma cells (64, 65) or the production of long-lived plasma cells which are challenging to target (66–68). T-cell driven education within the germinal center appears to be a requirement for the development of long-lived plasma cells, which gives rise to both lasting immunity against pathogens and chronic autoimmunity. Murine models of EBA have also demonstrated the presence of plasma cells with “intermediate” lifespans which contribute to autoantibody persistence (69). Alterations in cellular immune networks also contribute to maintained autoantibody production and disease chronicity: Increased Th1 and Th17 cytokines and chemokines have been reported in patients with pemphigus disorders (70, 71), BP (72, 73), and DH (74) and changes in Treg populations are also associated with AIBDs (75, 76), with Tregs thought to be protective against pathogenic autoantibody production (77, 78). Higher frequencies of Th17 cells secreting IL-21 have been reported in pemphigus lesions which form a tertiary lymph node like structure within the skin and promote autoantibody production (79). Further investigation into cellular contributions to autoantibody production may reveal additional therapeutic targets which can be used to control AIBDs.

## Autoantibody-induced Tissue Damage

**Figure 1:**
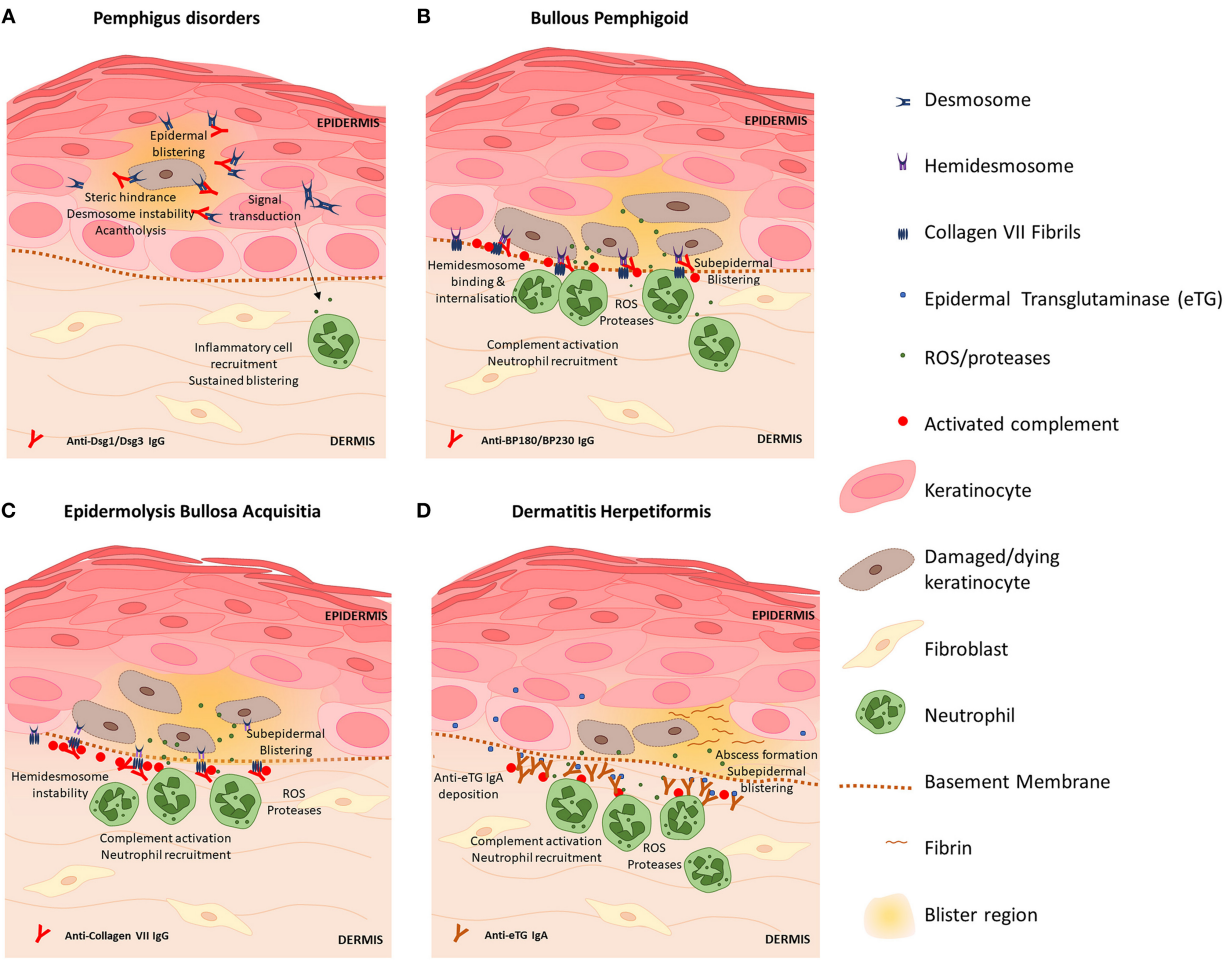
Blistering mechanisms of prototypic autoimmune skin blistering disorders result in skin barrier disruption. **(A)** Pemphigus disorders are caused by autoantibodies against desmoglein (Dsg) proteins Dsg1 and Dsg3. Binding of anti-Dsg destabilizes desmosomes to cause acantholysis of keratinocytes within the epidermis and triggers keratinocyte signal transduction events which promote inflammation, skin barrier disruption and further skin blistering. **(B)** Blisters in bullous pemphigoid are caused by anti-BP180 antibodies which bind hemidesmosomes on basal keratinocytes and trigger complement activation and inflammatory responses including ROS and protease release by neutrophils which directly kill keratinocytes. Skin barrier disruption and skin blistering is caused by keratinocyte death and sustained localized inflammation. **(C)** Epidermolysis bullosa acquisita is caused by anti-Collagen VII antibodies which bind fibrils that anchor hemidesmosomes to the basement membrane. Deposition of IgG induces complement activation via the classical pathway and activation of neutrophils. Basal keratinocytes sustain damage via action of neutrophil-derived ROS and proteases resulting in splitting at the dermal-epidermal junction and skin barrier disruption. **(D)** Dermatitis hepetiformis (DH) is caused by cross-reactive antibodies that bind epidermal transglutaminase (eTG). eTG is produced by keratinocytes and accumulates in the papillary dermis where it forms immunogenic immune complexes with anti-eTG IgA that trigger complement activation. Fibrin deposition and influx of leukocytes (which damage keratinocytes via release of ROS and proteases) cause the formation of neutrophilic abscesses which develop into fluid-filled subepidermal blisters that disrupt the intact skin barrier.

Autoimmune diseases of the skin are the result of pathological processes caused by autoantibodies against skin antigens. A number of common antigens targeted by disease-associated autoantibodies have been discovered, including antigens present on desmosomes, hemidesmosomes and proteins expressed by keratinocytes. Transfer of patient autoantibodies against Dsg 1 (80, 81) and Dsg 3 (82) and type VII collagen (83) is sufficient to cause skin barrier disruption and epidermal blistering in mice consistent with the associated clinical disease, thus autoantibodies are considered instrumental to pathogenesis in many AIBDs. Figure 1 provides a diagram summary of blistering mechanisms and skin barrier disruption in prototypic AIBDs pemphigus disorders, BP, EBA, and DH. Inflammation triggered by autoantibody binding recruits and activates a number of myeloid and lymphoid cell subsets that participate in blister development and damage to the skin barrier, however these vary with specific disease and clinical context. Previous reviews have in details described the preclinical animal studies and some clinical evidence elucidating the contributions of eosinophils (84, 85), mast cells (86, 87), Th17 (70, 88), and Treg (89–91) cells to AIBD's, and these may represent novel therapeutic targets. Here we focus on contributions of autoantibodies to skin barrier disruption in different disease settings.

Unraveling the precise mechanisms of autoantibody-induced pathology has been the focus of much research in recent years. Autoantibodies against Dsg proteins, as found in pemphigus diseases, were initially thought to interfere with Dsg-Dsg interactions in desmosomes by steric hindrance (92) however evidence for direct effects of Dsg-anti-Dsg binding on intracellular signaling events was later discovered, including the p38 mitogen-activated protein kinase pathway leading to acantholysis (93, 94). Anti-Dsg may also reduce the number of desmosomes by clustering Dsg on the cell surface and interfering with normal turnover of desmosomal proteins, thereby depleting desmosomes of Dsg (95) and promoting acantholysis. In more recent years, other autoantibody species (96–98) and non-Dsg interactions (99) have been identified as contributing to pemphigus pathology and have prompted the hypothesis that multiple pathways may act synergistically (100) to cause classical pemphigus disease pathology (101).

In BP, autoantibodies against BP180 and BP230 components of hemidesmosomes produce blistering at the dermal-epidermal junction. Activation of the complement cascade via classical and alternative pathways has been demonstrated to contribute to skin barrier disruption and BP pathology (102) and C3 deposition at the epidermal basement membrane is a common clinical finding (103). Complement activation induces inflammation and damages keratinocytes via cytotoxic action of neutrophils leading to skin blistering and barrier disruption (104). Though complement-independent mechanisms of BP pathology have been since described (105, 106) including direct activation of neutrophils via immune complex-FcγR binding, complement activation is still a prevalent target for novel BP therapeutics (103, 107). Autoreactive IgE and eosinophilia are common findings in BP patients and IgE immune complexes binding and activating eosinophils thereby contributing to blister formation has been shown in *in vitro* and *in vivo* studies (108), implicating eosinophils as potential therapeutic targets in BP.

Disease pathology in EBA is caused by predominantly IgG1 and IgG3 autoantibodies that bind Collagen VII within anchoring fibrils at the dermal-epidermal junction. As a rare disease, much of what is known about EBA pathogenesis has been elucidated with experimental models. Like BP, complement activation is considered key to EBA pathogenesis (109), however the alternative pathway appears to be the dominant pathway behind experimental EBA pathology (110). Activation of complement induces inflammation, leukocyte extraversion, complement activation and subsequent tissue damage and disruption of skin barrier via release of ROS and proteases from neutrophils and other myeloid cells. *Ex-vivo* studies of patient serum incubated with healthy skin and donor neutrophils exhibit loss of epidermal adherence, hence clearly indicating that antibodies mediate clinical EBA blistering via neutrophil activation (111). The role of T cells in EBA pathology is yet to be fully elucidated, however murine studies show that NKT and γδT cells likely amplify tissue damage in EBA via interaction with immune complexes and neutrophils (112).

DH is characterized by accumulation of ant-eTG IgA antibodies within the papillary dermis, however eTG is primarily expressed within superficial epidermal layers (113). It is hypothesized that eTG may be released into the blood, where interaction with IgA occurs in nearby dermal vessels; alternatively eTG may be deposited along the basement membrane as a result of trauma (54, 114), however further research is required to confirm these hypotheses. Following IgA deposition, papillary abscesses characterized by a neutrophilic infiltrate and fibrin accumulation form which develop into a split at the basement membrane and subepidermal blistering. Patients with DH show reduced levels of anti-inflammatory IL-10 and reduced Treg cell numbers in lesional skin compared to healthy skin (115) which indicates the role of Tregs in modulating local inflammatory responses in DH and represents an attractive therapeutic target.

## Targeted Approaches for Treatment of AIBDs

The use of different *in-vitro* systems and experimental animal models in recent years has significantly improved our understanding of AIBDs ultimately leading to novel diagnostic tools and differentiated therapeutic approaches for these disorders (6, 7, 75, 89, 109, 116–123). These approaches can be broadly grouped into: traditional and topical therapies; rituximab and intravenous immunoglobulins and other treatments in pre-clinical and clinical trials. In this review, we will provide a broad summary of traditional treatments and novel emerging therapies which are also summarized in Table 1.

**Table 1:** Current and emerging therapeutic approaches in autoimmune skin blistering diseases.

**Approach**	**Therapeutic agents**	**Stage of development**	**Disease**
Corticosteroids(oral or topical)	Prednisolone (12, 124, 125) Clobetasol propionate Dapsone, Sulfapyridine	First-line therapies	BP, PD, EBA, DH
Immuno-suppressant	Azathioprine (125) Mycophenolate mofetil Chlorambucil	Second-line therapies	BP, PD, EBA, DH
Antibody removal	Immunoadsorption Plasma exchange	Third-line therapies	BP, PD, EBA, DH
B-cell targeting	Anti-CD20 (Rituximab) (126) Anti-CD19 (68) Anti-CD22 (68) Antigen-mediated targeting (127) BCR signaling inhibition (128)	Second-line therapy Preclinical Preclinical Preclinical Phase III trials	BP, PD, EBA, DH PD PD PD PD
Immuno-modulatory	IVIG (129) Anti-TNFα (130) Anti-IL-17A (117) Anti-IL-5 (131, 132) Anti-Eotaxin-1 (133, 134) HSP-90 inhibition (135, 136) SYK inhibition (137) Vitamin D (122, 138)	Second-line therapy Off-label use Preclinical Completed Phase IIC ompleted Phase II Preclinical Preclinical Preclinical	BP, PD, EBA BP, PD, MMP BP BP BP EBA EBA PD, EBA
Complement targeting	Anti-C1s (107) Anti-C5 (103, 109) Anti-factor B (109) C5aR1 antagonist (109)	Preclinical Preclinical Preclinical Preclinical	BP EBA EBA EBA
Wound healing therapies	Anti-Flii (139)	Preclinical	EBA

*BCR, B-cell receptor; BP, Bullous pemphigoid; DH, dermatitis herpetiformis; EBA, epidermolysis bullosa acquisita; HSP, Heat-shock protein; IVIG, intravenous immunoglobulin; MMP, mucous membrane pemphigoid; PD, pemphigus disorders; SYK, spleen tyrosine kinase*.

First line therapies for AIBDs generally include systemic oral or intravenous corticosteroids (0.5–2.0 mg/kg/day) such as prednisolone (12, 29). Topical high potency corticosteroids such as clobetasol propionate have also been demonstrated to be efficient alternatives to oral prednisolone therapy in BP by reducing autoantibodies against BP180 and BP230 (2). For more severe patients unresponsive to topical therapy, oral prednisolone is combined with adjuvant immunosuppressant (azathioprine, mycophenolate, or rituximab). Unlike other AIBDs, oral corticosteroids do not normally have a dramatic effect in EBA and to date there are no randomized controlled trials providing level 1 evidence for EBA treatment (4). Milder forms of EBA and DH may respond well to topical steroids including dapsone or sulfapyridine, while rituximab has been reported to be effective for severe patients (12, 61, 124, 140, 141). Combining conventional systemic corticosteroids with rituximab treatment has also showed beneficial clinical outcomes in mucous membrane pemphigoid diseases (142).

Second line therapies include corticosteroid-sparing agents azathioprine, mycophenolate mofetil, or rituximab, which may be combined with intravenous immunoglobulin (IVIG) therapy. Clinically, IVIG administration has been shown to significantly improve BP disease symptoms for several weeks after infusion (129). Third line therapies are dependent on individual patient needs and include therapies in clinical trials: cyclophosphamide, IgE-targeted therapies, immunoadsorption to remove pathogenic autoantibodies, IVIG, methotrexate, and plasma exchange.

Preclinical studies have identified potential emerging therapies which target immunological mechanisms in AIBDs, which aim to reduce damaging inflammatory processes. Direct targeting of antibody-producing B cells is efficacious, as anti-CD20 antibody (Rituximab) has been successfully used in the clinic for multiple AIBDs, however not all patients respond equally. Targeting other B cell markers including anti-CD19 and anti-CD22, or antigen-specific B cell receptors may improve targeting of long-lived plasma cells which produce pathogenic autoantibody in patients that are refractory to rituximab. Therapies targeting components of the complement cascade have exhibited success in preclinical and clinical trials, however concerns with side effects associated with existing complement targeting therapies may limit the clinical utility of these approaches (143, 144). Previous studies have highlighted the roles of cytokines in mediating AIBD tissue damage and have identified multiple therapeutic targets including TNFα (130, 145), IL-5 (84), IL-17A(117), and IL-1 blockade (146) and administration of anti-inflammatory IL-10 (147). Immunomodulatory anti-cytokine therapies are in various stages of development, with existing TNF-α inhibitors showing efficacy in treating AIBDs and antibodies against IL-5 and eotaxin-1 having entered clinical trials for BP treatment (148–150). Like immunomodulatory therapies for other autoimmune diseases, these approaches are likely to be associated with known side effects of immunosuppression (151) thus further trials are needed to identify the safest and most efficacious dose regimens for specific diseases. Preclinical studies have identified cellular populations which may be exploited as therapeutic targets, such as Langerhans cells (152), dendritic cells (153), granulocytes (104, 111, 133, 154), and multiple T cell subsets including Th17 (117, 155), Treg (75), NKT (112), γδT (112), and CD8+ (156). Approaches to target specific immune cell functions are currently in preclinical development, including small molecule inhibition of spleen tyrosine kinase (137), an enzyme involves in proinflammatory Fc-receptor signal transduction in myeloid cells, and metabolites of Vitamin D which reduce myeloid cell activation and ROS production (122).

Understanding the mechanisms of autoantibody mediated tissue damage is critical in development of novel targeted therapies, however understanding the mechanisms behind resolution of blistering and inflammation in AIBDs can also offer insights into potential novel treatment modalities. Unresolved blistering can impact patient quality of life and increase risk of bleeding, infection and tumor development (157). Optimal healing is especially important for patients with extensive cutaneous blistering, or mucosal blistering which affects feeding, digestion and function of other organs (158, 159). Studies surrounding the role of Flightless protein (Flii) in skin blistering have revealed the first mechanism leading to the resolution of blistering and inflammation in antibody transfer induced EBA (8), and further research into similar pathways may reveal more potential therapeutic strategies where wound healing therapies may offer opportunities for decreasing the clinical symptoms associated with AIBDs.

In summary, there is a high unmet need for new targeted therapeutic approaches focussed on restoring the integrity of the skins' barrier 22and address both blistering mechanisms and clinical symptoms in systemic AIBDs. Innovative designs of randomized controlled trials using validated scales of assessment are needed to drive the development of novel therapeutic strategies for patients with AIBDs. Additionally, research efforts should focus on adapting immunomodulatory approaches that have been shown to be effective in other autoimmune diseases in order to target common pathogenic mechanisms and developing a better understanding of blister resolution and healing to improve patient symptoms.

## Conclusion

As highlighted in this review, the contribution of the skin barrier to the mechanisms underpinning autoimmunity has greatly improve our understanding of AIBDs. Development of novel targeted therapeutics restoring skin barrier function and homeostasis will lead to improved treatment of patients with AIBDs.

## Contribution to the Field Statement

Autoimmune skin blistering diseases are caused by pathogenic autoantibodies which trigger cellular, biochemical and immunological processes that disrupt the skin barrier and cause chronic blistering in patients. Understanding the mechanisms behind these processes has lead to the development of new targeted therapeutics which are in various stages of preclinical and clinical development. Current therapeutic approaches rely heavily on immunosuppressants and corticosteroids which are associated with adverse effects including risk of infection. Thus, new therapeutics are necessary to effectively control skin blistering and restore the skin barrier with fewer side effects. In this review, we highlight the different mechanisms behind autoimmune skin blistering disease development including initiation, maintenance and tissue damage. Additionally, we summarize current treatment and emerging therapeutics for autoimmune skin blistering diseases and highlight blistering mechanisms which may be exploited for development of novel targeted therapeutics.

## Author Contributions

All authors listed have made a substantial, direct and intellectual contribution to the work, and approved it for publication.

### Conflict of Interest Statement

The authors declare that the research was conducted in the absence of any commercial or financial relationships that could be construed as a potential conflict of interest.
